# Acute symptomatic seizures and status epilepticus in older adults: A narrative review focusing on management and outcomes

**DOI:** 10.3389/fneur.2022.954986

**Published:** 2022-08-26

**Authors:** Wan Yee Kong, Rohit Marawar

**Affiliations:** Department of Neurology, Wayne State University, Detroit, MI, United States

**Keywords:** acute symptomatic seizure, acute symptomatic status epilepticus, older adults, management, outcomes

## Abstract

A clear narrative of acute symptomatic seizures (ASyS) in older adults is lacking. Older adults (≥60 years) have the highest incidence of seizures of all age groups and necessitate a tailored approach. ASyS has a bimodal peak in infancy and old age (82.3–123.2/100,000/year after 65 years of age). ASyS can represent half of the new-onset seizures in older adults and can progress to acute symptomatic status epilepticus (ASySE) in 52–72% of the patients. Common etiologies for ASyS in older adults include acute stroke and metabolic disturbances. For ASySE, common etiologies are acute stroke and anoxic brain injury (ABI). Initial testing for ASyS should be consistent with the most common and urgent etiologies. A 20-min electroencephalogram (EEG) is less sensitive in older adults than in younger adults and might not help predict chronic epilepsy. The prolonged postictal phase is an additional challenge for acute management. Studies note that 30% of older adults with ASyS subsequently develop epilepsy. The risk of wrongly equating ASyS as the first seizure of epilepsy is higher in older adults due to the increased long-term challenges with chronic anti-seizure medication (ASM) treatment. Specific challenges to managing ASyS in older adults are related to their chronic comorbidities and polypharmacy. It is unclear if the prognosis of ASyS is dependent on the underlying etiology. Short-term mortality is 1.6 to 3.6 times higher than younger adults. ASySE has high short-term mortality, especially when it is secondary to acute stroke. An acute symptomatic etiology of ASySE had five times increased risk of short-term mortality compared to other types of etiology.

## Introduction

### Definition

The International League Against Epilepsy (ILAE) defines acute symptomatic seizures (ASyS) as seizures occurring in close temporal relationship with an acute central nervous system (CNS) insult of varying etiologies (1). The definition also includes seizures occurring in a preexisting background of epilepsy and fulfills other criteria for ASyS. Differentiating ASyS from unprovoked seizures is essential due to prognostic implications. ASyS differs from remote symptomatic seizures and progressive symptomatic seizures as the prognosis is different. For seizures to be considered ASyS, ILAE has proposed the following: seizure occurrence 1 week after stroke, head trauma, and anoxic brain injury (ABI), 1 day for metabolic causes, 7–48 h from the last alcoholic drink, and active intracranial infection or inflammation. Studies on optimal laboratory cutoff values to better delineate toxic–metabolic causes are lacking. Seizures due to anti-seizure medication (ASM) non-adherence are considered provoked seizures (1).

Older adults with epilepsy have the highest incidence of epilepsy of all age groups, with at least 25% of newly diagnosed seizures occurring after 60 years of age (2, 3). They constitute a separate treatment group compared to other adults due to the high incidence of comorbidities, associated polypharmacy, and age or disease-related changes in pharmacodynamics and pharmacokinetics (4).

There is a lack of consensus on who is considered an older adult in general and in those with seizures. We define older adults as ≥60 years of age for this review unless otherwise mentioned. Josephson et al. suggested using an age threshold of 65–70 years to define “elderly-onset epilepsy.” However, such a recommendation is lacking in ASyS or current older adults with “younger-onset epilepsy” (5).

### Epidemiology

Studies note that 34% of all afebrile seizures are ASyS (incidence 39 persons/100,000/year) (6). Men are more susceptible than women (42 vs. 27/100,000/year). Mirroring the incidence of epilepsy, ASyS also has two peaks, i.e., infancy and >74 years (7, 8). The rates start increasing gradually after 45 years of age: 55/100,000/year for 55–65 years; 82.3/100,000/year for 65–75 years; and 123.2/100,000/year for 75+ years. Older male adults are 1.6 to 2.6 more likely to have ASyS than older female adults (8).

According to Holt-Seitz et al. (9) ASyS represented half of the new-onset seizures in older adults. Nearly half were due to cerebrovascular disease (CVD), and 20% were due to metabolic disturbances (8). The rising incidence of CVD and metabolic disturbances with age (renal and hepatic dysfunction and diabetes) is likely responsible for the increased incidence of ASyS.

Acute symptomatic seizures represent 52–72% of cases of status epilepticus [SE; acute symptomatic status epilepticus (ASySE)] (10). ILAE definition of SE does not differentiate between the etiology of epilepsy and that of SE. For example, in a patient with a remote symptomatic cause of epilepsy such as CVD, the new SE might be triggered by a metabolic insult. This lack of differentiation is significant in older adults due to the potential for long-term polypharmacy (11). However, we assume that the definition of ASyS that includes “seizures occurring in a preexisting background of epilepsy” can be extrapolated to SE.

## Etiology

Cerebrovascular disease is the most common etiology of ASyS in older adults and accounts for 28–58% of ASyS (Table 1). ASyS are two times as likely with hemorrhagic CVD as compared to ischemic CVD (12). Cortical location predicts the risk of seizures after hemorrhagic and ischemic CVD (12). Regional ischemia leading to excitatory neurotransmitter release and local irritation due to acute mass effect from cerebral hemorrhage is the proposed pathophysiological mechanism underlying CVD-related AsyS (12). Other etiologies include metabolic (6–14%), traumatic brain injury (TBI) (7–10%), CNS infections (2–6%), and toxin-related (5–12%). Post-traumatic ASyS commonly occurs in the setting of acute subdural hematoma (13).

**Table 1:** Etiology of acute symptomatic seizures in older adults.

Proportion of ASyS cases by etiology for older adults^*^	**Traumatic brain injury**	**CVD**	**Central nervous system infection**	**Metabolic**	**Toxic**
Hauser et al. (14)	-	28%	-	-	-
DeLorenzo et al. (15)	-	35%	-	14%	11%
Ramsay et al. (3)	6.9%	35.8%	-	-	-
Sibia et al. (16)	4.40%	58.14%	6.16%	6.16%	5.28%
Annegers et al. (8)	10.2%	40.8%	2%	8.2%	11.6%

* Definition of older adults varies between age ≥ 60 years (3, 16) and ≥ 65 years (8, 14).

Acute stroke was responsible for 35% of ASySE in older adults (15). Other common etiologies in decreasing frequency were hypoxia (17%), metabolic disorders (14%), and alcohol-related (11%) (15).

## Clinical presentations and diagnostic workup

### Clinical presentations and differential diagnosis

Older adults can have atypical seizure presentations, such as epileptic aura, subtle confusion, aphasia, or prolonged postictal altered mental status. Non-motor manifestations, including somnolence and clumsiness, might be more common than convulsive seizures (17). Focal impaired awareness seizures originating from the frontal lobe are more common in the elderly than other types (18). Due to these atypical presentations, clinicians should consider seizures in the differential diagnosis of other common presentations in older adults, such as transient ischemic attacks, syncope, and falls.

Convulsive syncope is a seizure triggered by syncopal mechanisms caused by loss of vascular supply to the brain (19). The population incidence of convulsive syncope is unknown but is commonly seen in clinical practice. It can present as a transient myoclonic activity when the patient collapses with loss of consciousness. Surprisingly, head deviation, automatisms, and visual and auditory hallucinations, usually associated with focal onset seizures, are common (60–80%) in convulsive syncope (20) and can further lead to diagnostic dilemmas.

### Diagnostic testing

The American Academy of Neurology has published practice guidelines for the management of the first unprovoked seizure (21). However, a similar guideline is not available for first or recurrent provoked or ASyS.

#### Neuroimaging

The initial testing for ASyS in older adults should be consistent with the most common and urgent etiologies. Focal deficits with and after a seizure should prompt evaluation for CVD, including CT head to rule out acute intracranial hemorrhage and MRI brain for acute ischemic stroke. Neuroimaging is also helpful to evaluate underlying neoplastic or infectious processes. One study focusing on older adults has found CT head with the new-onset seizures to show an acute pathology in 35% of patients (9).

#### Electrophysiologic studies

Initial electroencephalogram (EEG) was abnormal in 73% (61 out of 84) of older adults with new-onset seizures. Notably, 64% showed focal slowing but only 39% had epileptiform discharges. However, these included patients with all types of seizures and were not limited to AsyS (9). Routine EEG was less sensitive in older adults and might not help distinguish an ASyS with no clear etiology from an unprovoked seizure with possible epilepsy (22). Prolonged EEGs or serial EEGs should be considered if the long-term risk of seizures and diagnosis of epilepsy is to be ascertained (23). The yield of detecting interictal epileptiform discharges increased by 50% when ambulatory EEG was performed, as compared to routine EEG (3). Prolonged EEG can help detect non-convulsive SE in older adults who present with acute confusional states (24). However, Rossetti et al. demonstrated that prolonged EEG, despite increasing seizure detection, did not change the outcomes as compared to routine EEG in critically ill patients without recent seizures (25).

For patients with suspected convulsive syncope, capturing the episode on EEG or prolonged cardiac electrophysiological tests (event monitor, loop recorder) might be required for a definitive diagnosis. A simultaneous tilt-table and EEG might help confirm the diagnosis in a small number of patients (26). Clinicians should direct management toward finding and treating the cause of syncope.

#### Laboratory studies

One of the commonest etiologies is an acute metabolic disturbance. Relevant testing, including serum glucose, electrolytes, renal, and hepatic function tests, would guide appropriate diagnosis and treatment. The other common etiologies are infections, i.e., systemic or CNS (meningitis and encephalitis). Fever and non-reactive leukocytosis should lead to further workup, including urine analysis, chest X-ray, and lumbar puncture. Toxicology testing, including alcohol level, should be performed at presentation. Alcohol (induced or withdrawal) is a common cause of ASyS and ASySE. Elevated serum alcohol levels can diagnose alcohol-induced ASyS. However, for the diagnosis of alcohol-withdrawal seizures, reliable history of consistent alcohol use and recent abstinence and low serum alcohol level are needed. A clinical institute withdrawal assessment for alcohol scale (CIWA-Ar) score can measure alcohol withdrawal symptoms and prompt appropriate prevention of alcohol-withdrawal seizures (27).

## Outcomes and management

### Outcomes

#### Short-term and long-term mortality of ASyS

The short-term risk of death with ASyS is high (around 20% in the first month post-ASyS). However, it is higher in older adults (28.4–40.5%) as compared to younger adults (11.2 vs. 17.7%). Thus, within the same population, the risk of death was 1.6 to 3.6 times for older adults (28). Similarly, short-term mortality due to ASySE is two times the rate in older adults compared to younger adults. This difference persists after excluding myoclonic SE due to ABI (29).

Despite increased short-term mortality of ASyS, studies show that long-term mortality is similar to ASyS and unprovoked seizures (30). ASyS does not predict functional outcomes at 6 months for patients with intracerebral hemorrhage in a prospective trial (31). However, these studies are not specific to older adults.

#### Mortality of ASyS by etiology

Cerebrovascular disease and ABI were the most common causes of ASyS in those with short-term mortality (28). Population-based studies fail to reveal if the increased risk of death is due to ASyS or the underlying etiology.

Similarly, ASySE in older adults has poor short-term outcomes (11). Acute symptomatic etiology was the commonest type of etiology for SE (52–58%) and also had a >6 times risk of poor outcome (death or new neurological impairment) as compared to other types of etiologies (11, 32). Two-thirds of patients with ASySE had a poor outcome, and 57% had inpatient mortality. CVD was the predominant acute symptomatic etiology (33). Similarly, Hui et al. (34) found that the acute symptomatic etiology of SE had five times increased risk of short-term mortality (49% mortality rate), with CVD the most common reason. In a study of convulsive SE in older adults, acute symptomatic etiology was the commonest cause, seen in 60% of the patients. Out of 33 patients, nine (27%) patients progressed to refractory SE. However, acute symptomatic etiology did not increase the likelihood of progressing to Refractory Status Epilepticus (RSE). CVD followed by metabolic disturbance was the most likely reason. None of the patients with CVD died. However, acute symptomatic etiology was associated with increased short-term mortality, seen in five out of six patients (35). Thus, in most studies, acute symptomatic etiology of ASySE and CVD as the specific etiology suggests poor outcomes in older adults.

Mortality in older adults with ASySE and acute ischemic CVD (39%) is much greater than in ischemic CVD alone (14%) or SE due to remote ischemic CVD (5%) (36). This finding demonstrates that the high mortality is due to “synergistic effects of SE and ischemic brain injury” (36). The increased mortality was not explained by the increased severity of CVD as measured by the size of the CVD. However, 63% of patients with acute ischemic CVD and 75% of patients with acute ischemic CVD + SE had negative CT or MRI imaging. This finding suggests that some of these patients possibly had a prolonged postictal focal deficit, which is commonly seen in older adults (37). It is unclear if the severity of ischemic CVD as measured by NIHSS is correlated with worse outcomes in concurrent SE (36).

There is conflicting evidence on whether acute stroke treatment with Tissue Plasminogen Activator (TPA) or thrombectomy increases the risk of ASyS. Two studies found that thrombolysis and thrombectomy increase the risk of poststroke seizures (38, 39) but were not replicated in a case–control study (40).

#### Risk of recurrence

The risk of subsequent unprovoked seizures after ASyS is about 30% and does not meet the criteria for a diagnosis of epilepsy (41, 42). Although ASyS has high short-term mortality, the risk of developing epilepsy is significantly lower than unprovoked or remote symptomatic seizures (43). A 10-year follow-up study found that the risk of future seizure recurrence is 3.3 times higher in ASySE as compared to ASyS. The risk is further modified by the underlying etiologies of ASyS, with ABI conferring the highest risk, followed by metabolic and structural causes (42). Thus, treatment with ASM in older adults with ASySE might be warranted, but not with ASyS.

It is unclear if seizure recurrence risk after ASyS is more in older adults than in young adults or if there is risk stratification with different etiologies. More than three seizures at presentation and epileptiform activity on initial EEG were predictors of subsequent unprovoked seizures and epilepsy (9).

A meta-analysis noted that ASyS increases the risk of poststroke epilepsy. Although this finding is not specific to older adults, most CVDs occur in older adults, so the conclusion can potentially be extrapolated to the older adult cohort (44). ASyS due to CVD carries the highest weight in the SeLECT score that provides risk-stratification of postischemic stroke epilepsy (45). A large majority of participants in the SeLECT study were ≥60 years and provided ample evidence of seizure risk in this cohort. Similarly, ASyS is a risk factor for the development of epilepsy in posthemorrhagic CVD as measured by the CAVE score (46).

For all adults with TBI, the risk of recurrent seizures increases if seizures occur within 1 week of injury, with severe and penetrating injury, prolonged loss of consciousness, intracerebral hemorrhage, and subdural hemorrhage requiring surgical evacuation (49).

Physicians often encounter outpatient scenarios where patients are inappropriately started on ASMs due to ASyS in an acute setting. The abovementioned risk factors in addition to patient comfort and projected consequences of a seizure (even if low risk) such as injuries in job setting and loss of driving privileges should be considered to decide continuation vs. gradual weaning off of the ASM.

### Management

Initial treatment of ASyS is directed toward the management of underlying etiologies. Patients with ischemic CVD presenting within a thrombolytic or endovascular window should receive cerebral revascularization treatment accordingly. In the 2019 American Heart Association guideline for the early management of ischemic CVD, IV Alteplase is reasonable in patients presenting with seizures at symptom onset if the residual deficits are attributed to CVD (50).

For patients with metabolic disturbances, correction of electrolyte and glucose disturbances is the most effective management for ASyS. Due to polypharmacy and multiple comorbidities, older adults may be prone to these disturbances. Investigating and treating underlying metabolic etiologies long-term is crucial to preventing future episodes. ASM is indicated to prevent recurrent seizures if there is an expected delay in correcting some metabolic derangement and hypoxia. Older adults are also prone to systemic and CNS infections due to immunosenescence (51, 52). New-onset seizures, especially in the setting of encephalopathy, should prompt early investigation and treatment of these infections. For patients with toxin ingestion or medication-induced (e.g., digoxin) ASyS, the culprit drug or toxin cessation and antidote administration (if available) are the most effective ways to treat seizures. Drug cessation and close monitoring of levels can help normalize epileptiform activity on EEG (53).

Anti-seizure medication is indicated for ASySE and might be necessary for treating ASyS that persist despite treatment of the underlying cause. Unfortunately, studies to guide the long-term management of ASM in the setting of ASyS are lacking. Patients who have interictal epileptiform discharges and persistent structural abnormalities or who present with SE are at a higher risk of developing epilepsy. The indication and duration of ASM in ASyS differ depending on the underlying etiology (Table 2) (43, 48, 50, 54–56). A 1–3 month duration has been proposed for short-term ASM use (43). For patients with ASyS due to posterior reversible encephalopathy syndrome (PRES), 3 months of ASM treatment have been proposed (57).

**Table 2:** Antiseizure management for acute symptomatic seizures (ASyS).

**Management of ASyS**	**Traumatic brain injury**	**Cerebro** **vascular** **disease**	**Central nervous system infection**	**Neoplastic**	**Metabolic**	**Alcohol**
Primary prophylaxis	Yes	No	No	No	No	Yes^a^
Short-term ASM	Yes	Possible^b^	Yes	Possible^d^	Possible^e^	Yes
Long-term ASM	Possible^e^	Possible^c^	Possible^d^	Possible^d^	No	No

a ASM prophylaxis can be considered for severe alcohol withdrawal.

b In selected cases such as ischemic stroke with hemodynamically relevant stenosis, brain edema, or vasospasms after subarachnoid hemorrhage (47).

c ASM can be considered in ischemic CVD based on SeLECT score (45) and in hemorrhagic CVD based on CAVE score (46).

d ASM should be continued if there are persistent structural abnormalities due to neoplasm or CNS infection or stroke.

d ASM can be considered if there is a delay in metabolic derangement correction.

e In patients with SDH requiring surgical evacuation, multiple brain contusions, early seizures, and dural penetrating injuries (48).

Primary seizure prophylaxis is not recommended for patients with acute ischemic CVD(50)or with intracranial neoplasm (54, 55). The Brain Trauma Foundation recommends 7-day anti-seizure medication prophylaxis after a TBI (56). Benzodiazepines or phenobarbital may be considered for seizure prophylaxis in severe alcohol withdrawal as determined by the CIWA-Ar score (43).

Few observational studies have found preventive benefits against ASyS with statins (58, 59). However, prospective, randomized studies are lacking to strongly recommend statin use at this time. The management of ASyS is summarized in Table 2.

#### Choice of ASM

Choosing an ASM in the setting of ASyS in older adults is challenging. Physicians need to consider underlying etiologies of ASyS, concurrent medications, comorbidities, and altered drug metabolism.

Strong hepatic enzyme inducers such as carbamazepine, phenytoin, and phenobarbital and enzyme inhibitor such as valproic acid should be used with caution due to potential interaction with multiple drugs used in older adults (60). Similarly, these ASMs should be avoided in patients with neoplasms due to drug–drug interaction with chemotherapeutic agents (61). They are also known to be atherogenic due to the potential of increasing serum cholesterol and, hence, are a suboptimal choice for poststroke seizures (61), a common reason for ASyS in older adults. Levetiracetam can improve cognitive outcomes after hemorrhagic CVD (62). It is comparable with phenytoin for the efficacy of primary and secondary seizure prophylaxis in TBI (43).

Patients with metabolic derangement secondary to renal or hepatic dysfunction require further consideration. Hepatically and renally metabolized or cleared ASM should be avoided or dose adjusted accordingly. Older adults can have physiologically decreased creatinine clearance (CL_CR_). Specifically, for levetiracetam, dose adjustment is required for CL_CR_ < 80 ml/min/1.73 m^2^ (63). Similarly, a maximum dose of 300 mg/day is recommended for lacosamide for severe renal impairment (CL_CR_ < 30 ml/min) (64). An additional dose of up to 50% once a day on baseline is recommended for levetiracetam and lacosamide in end-stage renal disease after hemodialysis on dialysis days (63, 64).

Randomized trials on ASMs targeting older adults are limited. However, two systematic reviews and meta-analyses suggest that lamotrigine, levetiracetam, and lacosamide are first-choice ASMs based on their high efficacy and reasonable tolerance in older adults (65, 66). Overall, older adults require lower doses to be seizure-free. For example, a median lamotrigine daily dose of 100 mg was sufficient for seizure freedom in many patients (67). A reasonable strategy for ASMs in older adults is “Start low, go slow, and stay low.”

#### Special considerations in the management of ASyS in the older adults

Differentiating between ASyS and unprovoked seizures can be challenging. However, the diagnostic dilemma has worse outcomes in older adults. The risk outcome of diagnosing a seizure as “unprovoked” when it was ASyS is more with older adults as they might be started on unnecessary chronic ASMs. This action can add to the medication burden in patients with polypharmacy (68). ASMs are frequently associated with adverse effects in older adults, including falls, cognitive impairment, and long-term complications like osteoporosis (69). In addition, they increase the risk of hospitalization (70).

Older adults can have prolonged postictal symptoms, including altered mental status, lasting hours to days (37). Thus, even if the etiology is known to be acute symptomatic, this prolonged postictal state could be confused with non-convulsive SE and could lead to needless treatment with benzodiazepines, ASMs, and anesthetic medications. This intervention further leads to an increased risk of intubation and increased length of stay which are known to have worse short-term and long-term outcomes (11). Obtaining an emergent EEG is of paramount importance in these patients to distinguish the postictal state from non-convulsive SE. Even in patients with established epilepsy, failure to distinguish between ASyS and unprovoked seizures will lead to an unnecessary increase in the dose of baseline ASMs.

## Conclusion

Diagnosis and management of ASyS is an emerging field with many unanswered questions in older adults, including patient selection and risk factors for primary prophylaxis, short-term and long-term outcomes, and ASM management. Some novel clinical endeavors such as post-ASyS clinics and the multicenter Post-Acute Symptomatic Seizure Investigation and Outcomes Network (PASSION) project will help to further clarify many unknowns in this field (71).

## Author contributions

WK and RM were responsible for the conception and design of the paper, drafting, critical revision, and final approval of the article to be published.

## Conflict of interest

RM has received funding for investigator-initiated trial from Eisai Co. The remaining author declares that the research was conducted in the absence of any commercial or financial relationships that could be construed as a potential conflict of interest.

## Publisher's note

All claims expressed in this article are solely those of the authors and do not necessarily represent those of their affiliated organizations, or those of the publisher, the editors and the reviewers. Any product that may be evaluated in this article, or claim that may be made by its manufacturer, is not guaranteed or endorsed by the publisher.
